# Use of Non-Amplified RNA Samples for Microarray Analysis of Gene Expression

**DOI:** 10.1371/journal.pone.0031397

**Published:** 2012-02-15

**Authors:** Hiroko Sudo, Atsuko Mizoguchi, Junpei Kawauchi, Hideo Akiyama, Satoko Takizawa

**Affiliations:** New Frontiers Research Laboratories, Toray Industries, Inc., Kamakura, Kanagawa, Japan; Institute for Systems Biology, United States of America

## Abstract

Demand for high quality gene expression data has driven the development of revolutionary microarray technologies. The quality of the data is affected by the performance of the microarray platform as well as how the nucleic acid targets are prepared. The most common method for target nucleic acid preparation includes *in vitro* transcription amplification of the sample RNA. Although this method requires a small amount of starting material and is reported to have high reproducibility, there are also technical disadvantages such as amplification bias and the long, laborious protocol. Using RNA derived from human brain, breast and colon, we demonstrate that a non-amplification method, which was previously shown to be inferior, could be transformed to a highly quantitative method with a dynamic range of five orders of magnitude. Furthermore, the correlation coefficient calculated by comparing microarray assays using non-amplified samples with qRT-PCR assays was approximately 0.9, a value much higher than when samples were prepared using amplification methods. Our results were also compared with data from various microarray platforms studied in the MicroArray Quality Control (MAQC) project. In combination with micro-columnar 3D-Gene™ microarray, this non-amplification method is applicable to a variety of genetic analyses, including biomarker screening and diagnostic tests for cancer.

## Introduction

Microarray permits the simultaneous analysis of hundreds of thousands of genes in a relatively short time using a small amount of sample material. The quality of expression data, however, is affected not only by the microarray performance, but also by how the nucleic acid targets are prepared. The most common method for nucleic acid target preparation includes *in vitro* transcription amplification of the sample RNA, which allows the initial amount of starting material to be in the nano- or pico-gram range [1]–[3]. The amplification method is also reported to show high reproducibility and strong correlation with qRT-PCR [4]. One drawback of the amplification method is that it is a long and intensive process, which also leads to increased labor costs. Furthermore, the complicated protocol is difficult to adapt for diagnostic or medical testing applications, which demand a quick and simple process. However, perhaps the most concerning issue regarding the amplification method is data accuracy. Amplification bias that is generated during the *in vitro* transcription step may distort the quantitative measurement of transcript abundance. Accurate detection of gene transcript abundance as well as of differential expression ratios is critical. Failure to accurately detect these may have serious consequences, particularly when the results obtained are applied to medical tests or clinical diagnoses.

A sample preparation protocol that does not require RNA amplification exists and has been used since the beginning of the microarray technology era [5]. However, with the increase in available amplification methods, the non-amplification protocol has been largely replaced, likely due to its requirement for a large amount of RNA starting material. Currently, most microarray manufacturers including Affymetrix [6]–[8], Agilent [9]–[11] and Illumina [12] recommend using amplified RNA samples for gene expression analysis to minimize the amount of starting RNA required.

We previously developed a novel microarray, 3D-Gene™, which features a micro-columnar structure composed of black resin substrate and a bead-agitation technique. This achieves low background noise, enhanced signal intensity, and high reproducibility in detecting gene expression profiles [13]. The system has demonstrated high sensitivity in microRNA detection [14] as well as in multiplex single-nucleotide polymorphism (SNP) detection [15].

In this study, we used the 3D-Gene™ microarray platform and compared samples prepared using either a conventional amplification protocol or a non-amplification protocol. Samples from the non-amplification procedure had higher quantitative accuracy than those from the amplification method, with competitive detection power and reproducibility. Our results suggest that when combined with micro-columnar 3D-Gene™ microarray, the non-amplification method for nucleic acid preparation is a reliable and practical technique for gene expression profiling.

## Results

### Quantitative and qualitative reproducibility and concordance

**Figure 1:**
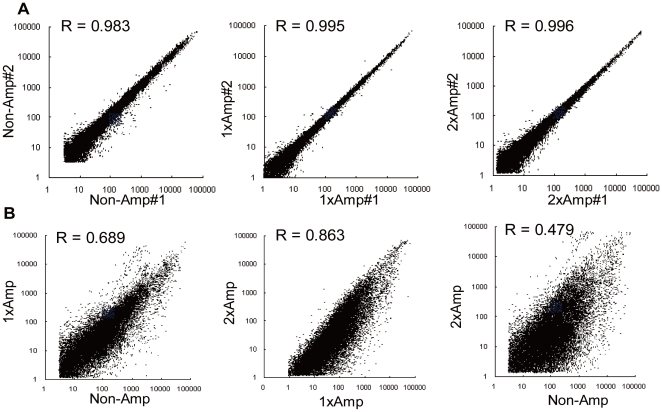
Intra- and inter-method gene expression comparison using UHRR samples. **1A:** Intra-method comparisons of UHRR assayed by the non-amplification (Non-Amp), the 1-round amplification (1xAmp), and the 2-round amplification (2xAmp) method. **1B:** Inter-method comparisons between the non- and 1-round amplification methods, between the 1- and the 2-round amplification methods, and between the non- and 2-round amplification methods. The scatter plots compare the logarithmic scale (base 10) signal intensities expressed by each gene from duplicate microarray experiments. Pearson's correlation coefficient (R) is in the top left corner of each plot.

To assess the effect of amplification during sample RNA preprocessing, we first examined the reproducibility of quantitative signal values and qualitative detection calls detected by non-amplification, 1-round amplification or 2-round amplification methods. Duplicate samples of Universal Human Reference RNA (UHRR) were prepared by each of the three methods and analyzed by microarray. The intra-method reproducibility was similar among the three methods with a slight decrease in Pearson's correlation for the non-amplification method (Fig. 1A). The proportions of genes in which signal intensity values were detected within a range of 2-fold change in the duplicate experiments were 98.1%, 99.2% and 97.9% for the non-, 1-round and 2-round amplification methods, respectively. The proportions of undetected genes were in a similar order: 7.5%, 3.8% and 10.8% of a total of 24,267 genes for the non-, 1-round, and 2-round amplification methods. In contrast to the high intra-method reproducibility, correlation values from inter-method comparisons showed significantly reduced reproducibility. The Pearson's correlation value was 0.689 between the non- and 1-round amplification, 0.863 between 1- and 2-round amplification and 0.479 between the non- and 2-round amplification methods (Fig. 1B).

**Figure 2:**
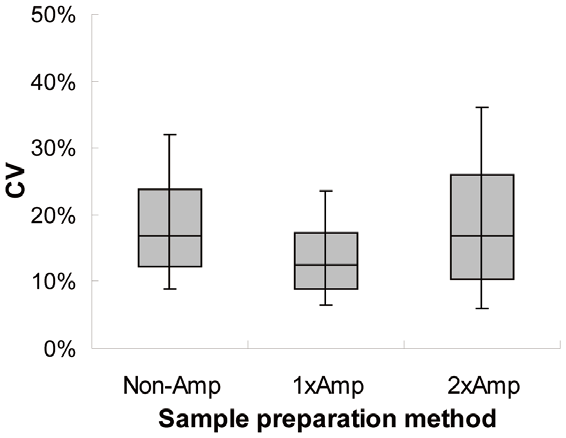
Intra-method reproducibility of gene expression signals among replicates. HURR was assayed in replicates by the non-amplification (Non-Amp), the 1-round amplification (1xAmp) or the 2-round amplification (2xAmp) method and detected by microarray. A total of 11,365 genes present in both 3D-Gene™ microarray and the MAQC probe set were used for the calculation. The distributions of the coefficient of variation (CV) are presented as boxes: the bottom and the top of the box represent the 1^st^ and 3^rd^ quartile whereas the band near the middle represents the median. The whiskers represent the 10^th^ and 90^th^ percentiles.

The reproducibility was also calculated using the coefficient of variation (CV) of the signal intensity from replicates of the UHRR sample. To compare the CV values published in MAQC [4], 11,365 gene probes commonly present in both the 3D-Gene™ microarray and the probe set selected from the MAQC study were used for the calculation. Only genes that were detected in at least three of the five (60%) sample replicates for the non- and 1-round amplification method, or at least two of the three (67%) replicates for the 2-round amplification method were included in the CV calculation. The numbers of detected genes meeting these criteria were 10,012, 10,567 and 9,437 for the non-, 1- and 2-round amplification methods, respectively. The replicate median CV±standard deviation were 0.17±0.10, 0.13±0.08, 0.17±0.13 for the non-, 1- and 2-round amplification methods, respectively (Fig. 2).

**Figure 3:**
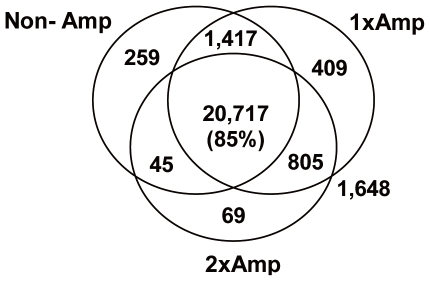
Qualitative concordance of detected genes by three sample preparation methods. Among 24,267 gene spots on the microarray used, genes that were detected in at least 60% of the sample replicates were used to create the Venn diagram.

Next, we evaluated the qualitative concordance of the detected genes between the three sample preparation methods. More than 85% (20,717 of 24,267) genes were commonly detected by the three preparation methods. 1.1% (259 of 24,267) genes were uniquely detected by the non-amplification method while 1.7% (409 of 24,267) genes were uniquely detected by the 1-round amplification method (Fig. 3). It should be noted that the number of overlapping genes detected by both the non- and 1-round amplification methods (1,417 genes) is much larger than the number of overlapping genes detected by both the non- and 2-round amplification methods (45 genes). This suggests that multiple rounds of amplification would create further diversion in the expression profiles from the original unamplified profile.

### Relative Accuracy

To assess the relative accuracy of the microarray data, an alternative technology was used to measure gene expression. Four different human RNA samples were processed by the non-, 1- or 2-round amplification method and analyzed by microarrays. The results were compared with data obtained from qRT-PCR assays analyzing the same samples. For this comparison, we selected 42 genes based on our preliminary study, 25 of which (#1–25 in Table S1) were randomly selected and 17 of which (#26–42 in Table S1) were selected due to their susceptibility to amplification bias.

**Figure 4:**
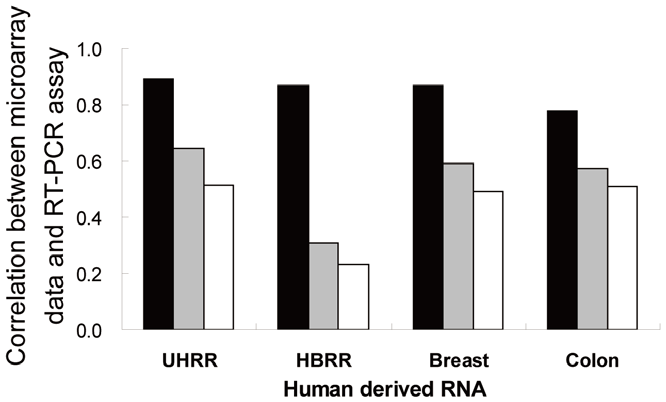
Spearman's rank correlation between the microarray assay and qRT-PCR assay. UHRR, HBRR, breast, and colon RNA derived from human tissue or cells were processed by the non-amplification (black bar), the 1-round amplification (grey bar), or the 2-round amplification (white bar) method, analyzed by microarray. The obtained PolR2A-normalized signal intensity (log_2_) of the 42 genes (Table S1) was compared with ΔCt values of the same samples analyzed by qRT-PCR assays.

We found that the microarray data processed using the non-amplification method had the highest Spearman's correlation (ρ = 0.84–0.93) with the qRT-PCR data for all four RNA samples (Fig. 4). Correlation with the qRT-PCR data decreased as the rounds of amplification increased (ρ = 0.44–0.74 for 1-round amplification and ρ = 0.29–0.62 for 2-round amplification). This indicates that the amplification process during sample preparation in fact reduces the relative accuracy of the microarray data.

**Figure 5:**
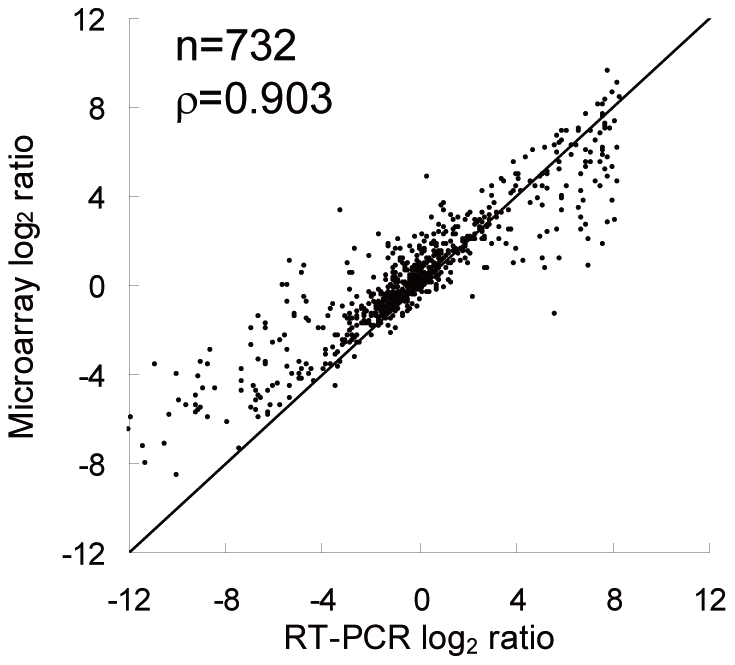
Correlation between microarray and MAQC qRT-PCR assay. The scatter plot compares the log_2_ differential expression ratios of HBRR versus UHRR obtained from the non-amplification method on microarray and from qRT-PCR assays published in MAQC [4]. The number of genes compared (n) and the Spearman's correlation (ρ) are listed in the top left corner of the plot. The diagonal line indicates the ideal y = x line.

The differential expression data obtained from Human Brain Reference RNA (HBRR) and UHRR using the non-amplification method were further compared to the qRT-PCR assay values published in MAQC [4]. Out of 996 genes present both on the microarray platform used in this study and the published qRT-PCR assay, 732 genes were detected in both HBRR and UHRR on the microarray and the qRT-PCR assay (Fig. 5). The correlation between the non-amplification method on microarray and the qRT-PCR assay was 0.903. By comparison, other microarray platforms published in MAQC report the correlation values of 0.839–0.905 [4].

## Discussion

Comprehensive analyses of gene expression profiles using high-throughput technologies such as microarray provide valuable information useful for the elucidation of molecular mechanisms and cellular functions. In many gene expression assays, the target nucleic acids undergo preprocessing before microarray detection. The most commonly used target RNA processing methods involve linear amplification by *in vitro* transcription to complementary RNA using T7 RNA polymerase [16]. Although amplification is critical for some studies in which sample material is limited, it is known that the amplification process can introduce bias or distort the initial transcript abundance. It is naturally drawn that direct analysis of cDNA obtained by reverse transcription of RNA samples would avoid such bias and therefore provide better fidelity in the detection of transcript abundance. Using an unamplified RNA sample, we previously reported the satisfactory performance of 3D-Gene™ microarray we had developed [13]; however, the protocol used in the previous study showed relatively small fold-changes in gene expression when samples derived from two different tissues (human brain and liver) were analyzed.

In this study, we combined the 3D-Gene™ microarray platform with an improved non-amplification method for target RNA preparation and demonstrated competitive reproducibility and detection coverage. The CV for the 1-round amplification method was similar to the median values (5–15%) reported in the MAQC [4], whereas the CV values for the non- and 2-round amplification method were slightly higher (17%). Importantly, the non-amplification method showed better accuracy than the either amplification method in all four types of human-derived tissue or cellular RNA tested (Fig. 4). In Figure 1, we show that rounds of amplification introduce the artificial diversity and compromise the accuracy in measured transcript abundance. This is further supported by strong correlation with qRT-PCR assay (Fig. 4).

We further validated the accuracy of the non-amplification method by comparing the data obtained with qRT-PCR assays published by MAQC [4]. Among the microarrays tested in the MAQC, Eppendorf and National Cancer Institute (NCI) prepared the RNA samples without using *in vitro* transcription amplification. These are unique microarray platforms because Eppendorf is a low-density array characterized by a unique data structure and NCI uses dual-color labeling which defines the signal background differently. Therefore, we believe that the results obtained from these microarray platforms do not accurately evaluate true potential of the non-amplification method. Herein, by utilizing the mono-color labeling method and a comprehensive microarray platform, we reevaluated the non-amplification method. Our non-amplification method detected 732 out of 996 genes, the largest detection coverage as the mono-color labeling method. In addition, the correlation of our non-amplification method on microarray with the qRT-PCR assays was one of the highest in the seven platforms studied in MAQC [4].

Furthermore, using the same microarray platform and the same labeling method, we directly compared the effect of the target amplification on detection accuracy. This is indirectly estimated by correlation with qRT-PCR, the current golden standard for gene expression measurements. We found dramatic decreases in the correlation coefficients as rounds of target amplification increased. It should be noted that 17 of the 42 genes studied were pre-selected due to their potential for amplification bias; therefore, the effect of amplification bias might be less dramatic if the target genes are expanded to include whole transcriptome analysis. However, if genes that are particularly susceptible to amplification bias are selected as targeted biomarkers in focused microarrays for diagnostic use, the consequence of distorted measurements could be devastating. It is imperative to choose a method that reflects true transcript abundance, especially in clinical settings and diagnostic tests.

It has been reported that gene expression data obtained from 1-round amplified RNA is substantially different from data obtained from 2-round amplified RNA. Croner *et al.* performed unsupervised hierarchical cluster analyses that include all 22,283 probe sets from the Affymetrix Gene chip and separated 1-round amplified samples from 2-round amplified samples [17]. It is also reported that the gene expression ratios of two samples (such as treated versus untreated) tend to decrease when the amplification procedure is used. Gilbert et al. reported that half of differentially expressed candidate genes were undetectable using the recommended amplification procedure, thus distorting the true proportional differences [18]. In an effort to explain the observed bias, this group investigated T7 *in vitro* transcription reaction kinetics and discovered that aRNA production was linear only for 40 min of the first round and for 50 min of the second round amplification. This is followed by a non-linear phase, which introduces the bias that leads to inaccuracies in transcript abundance [18]. However, many *in vitro* transcription protocols (including Arcturus™ RiboAmp® HS PLUS, Ambion's MessageAmp™ II and Epicentre's TargetAmp™) recommend 4–14 hours of the incubation, thereby providing idling time for non-linear RNA amplification and subsequent bias. Our current study further supports the conclusion of Gilbert et al. that the non-amplification method generates larger gene expression ratios and thus more differentially expressed genes than amplification methods do.

Other explanations of the amplification bias have been hypothesized. Kerkhoven et al. reported that T7-based linear amplification bias is caused by the 3′ spacer sequence of the amplified RNA, which excessively binds to probes that share similar sequence with the T7 motifs [19]. It has also been reported that amplification bias is caused by molecular features of the affected RNA sequences, including the position within the gene, the GC content, hairpin numbers, and the length of poly-A stretches [20]–[22].

We assume that the better accuracy obtained by the non-amplification method compared to the 1-round amplification method in this study is due to the absence of these molecular hindrances. Additionally, we attribute our improved results to two factors that enhanced signal intensity: satisfactory performance of our microarray platform [13] and use of a signal amplifier such as dendrimer [23] in the sample labeling process.

Another advantage of the non-amplification method is the shorter processing time (currently several hours) compared to amplification methods (1.5 days). This directly translates to lower labor costs. Finally, the simplicity of the procedure is also advantageous if the system is to be automated, which is necessary for applying the methods in a clinical setting.

Despite our current improvements, the non-amplification method as presented can be further refined. Nearly all of the non-amplification data presented herein were produced using 10 µg of total RNA. Similar reproducibility (R = 0.984) was observed for the non-amplification method when the amount of RNA was reduced from 10 µg to 3 µg (data not shown). This quantity is still too large for specific study settings, such as small tissue biopsies or laser micro-dissection samples. However, this method can be applied to researches that have less stringency in sample limitations, including studies that involve cell culture or large surgical specimens.

One way to reduce the input RNA quantity while maintaining the reaction concentration is to engineer a device that decreases the hybridization reaction volume. The hybridization reaction volume used in this study was 210 µl. If the hybridization reaction volume is decreased to 10 µl, which is quite feasible, the amount of sample RNA could be 20-fold smaller or ∼150 ng. Additionally, if the analysis is not for the whole transcriptome but targeted to a limited number of genes, the size of the system can be further reduced. These allow the method to be more accessible for various studies, including diagnostic testing. For example, MammaPrint®, an *in vitro* diagnostic test based on gene expression microarray requires 200 ng total RNA extracted from biopsy or surgical specimens [24]. Further technical developments that reduce the sample quantity are necessary before the non-amplification method can be used in a wide range of clinical research and tests.

We have demonstrated that our RNA preprocessing method that does not involve sample amplification is accurate in transcript measurement, thus providing reliable gene expression profiles. When combined with micro-columnar 3D-Gene™ microarray technology, this non-amplification method can be employed for a variety of applications, including clinical diagnoses and medical tests.

## Materials and Methods

### RNA sample

The following commercially available total RNA was used in this study: Universal Human Reference RNA (UHRR, Stratagene #740000), Human Brain Reference RNA (HBRR, Applied Biosystems #AM6051), human breast total RNA (Applied Biosystems #AM6952) and human colon total RNA (Applied Biosystems #AM7986).

### Non-amplified sample preparation and hybridization to microarray

10 µg total RNA was used unless otherwise indicated. The RNA and 2 µl Anchored Oligo dT_20_ (2.5 µg/µl Invitrogen #55117) were added to nuclease-free H_2_O to a final volume of 20 µl and incubated at 80°C for 10 min, then immediately placed on ice for 3 min. The RNA was reverse transcribed using SuperScript®II kit (Invitrogen #18064-014) with 4 µl of 0.16 mM dNTP mixture and 2 µl Biotin-16-dUTP (1 mM, Roche Diagnostic #1093070), incubated at 42°C for 2 hrs. Nuclease-free H_2_O (156 µl) and 4 µl 1.0 M NaOH were added to the cDNA product and incubated at 37°C for 10 min. For alkaline neutralization, 20 µl 1 M Tris-HCl (pH 6.8) was added. The cDNA was purified using DNA Clean & Concentrator-5 columns (Zymo, #D4013). The concentration of the obtained cDNA was measured using a spectrophotometer (Nanodrop ND-1000 version 3.0.0., NanoDrop Technologies).

The cDNA was mixed with 21 µg of each human and yeast non-coding nucleic acid sequence as a blocking agent and nuclease-free H_2_O was added to a final volume of 47.3 µl. The mixture was denatured at 95°C for 5 min then immediately placed on ice for 3 min. This cDNA mixture was added to 162.7 µl of 42°C pre-warmed hybridization buffer which includes formamide and SDS. The hybridization mixture was vacuumed at 0.01 MPa for 20 min, incubated at 42°C for 3 min and applied to the 3D-Gene™ Human Oligo chip 25 k (Toray Industries, Inc. TRT-XR125). The microarrays were hybridized on a 250 rpm shaker at 42°C for 16 hrs as recommended by the manufacturer.

The microarrays were washed as described in the product manual (v1.06). The spin-dried microarray underwent the post-labeling procedure. First, 2.0 µl UltraAmp™ SA (40) Oyster-650 (Genisphere Inc. #SA0460) and 2.0 µl UltraAmp™ SA (40) Nucleic Acid Blocker (Genisphere Inc. #SA04BLK) were mixed with 125 µl 2X post-labeling buffer, as per the manufacturer's instructions and H_2_O was added to a final volume of 250 µl. This post-labeling reagent mixture was incubated at 4°C for 5 min and applied to the hybridized microarray, which was attached and enclosed with a plastic cover with double-sided adhesive tape. The holes in the microarray were sealed with tape and incubated at 4°C for 30 min in dark. After the labeling reaction, the plastic cover was removed from the microarray and it was washed as described in the product manual (v1.06). The microarray was then spin dried, followed by the image scanning.

### Amplified sample preparation

For both 1- and 2-round amplification methods, sample preparation, hybridization, and washing were performed according to the 3D-Gene™ Human Oligo chip 25K manual (v1.06). The linear amplification of RNA samples was performed using the Ambion's Amino Allyl MessageAmp™ II aRNA Amplification Kit (Ambion, #AM1753), per the manufacturer's instructions.

### Image scanning and analysis of gene expression

Microarrays were scanned using ScanArray® Lite (Perkin Elmer) at an excitation wavelength of 635 nm with 100% laser power. The photomultiplier settings of the red channel were manually adjusted according to procedures recommended by the manufacturer. The obtained images were numerated by GenePix® Pro6.0 (Molecular Device) and the spot intensity was calculated by taking the median intensity of the foreground signals. The background signal intensity is derived by taking the mean signal intensity of the blank spots that excludes the top and bottom 5% signal intensities. The detected spots were defined as those that had signal intensity above the 95% upper confidence interval of the background signal intensity. For detected spots, their signal intensities were determined after subtracting with the mean background signal. For data comparison, the background-subtracted signal intensity was normalized using global normalization in which the median from each microarray was used.

All microarray data from this study are complaint with Minimum Information About a Microarray Experiment (MIAME) and is publicly available through the NCBI's Gene Expression Omnibus (GEO) database (http://www.ncbi.nlm.nih.gov/projects/geo/) under the series record GSE30945. The accession numbers are listed in Table S2 (Supporting Information).

To assess the signal reproducibility of each sample preparation method, the Pearson's correlation coefficient (R) and the coefficient of variation (CV) of the signal were calculated. The CV for each mRNA assessment was calculated by the formula CV% = (standard deviation/mean)×100.

### Real-Time PCR

The relative accuracy of the data obtained by microarray was evaluated by comparing them with data obtained from an alternative detection technology. TaqMan® assays (Applied Biosystems), one of the most accurate methods in the quantitative real-time PCR (qRT-PCR) system, were used in this assessment, per the manufacturer's instructions. Forty-two genes were used for this comparison (Table S1). qRT-PCR was performed for each gene in quadruplicate and the mean was calculated as the threshold cycle (Ct) value. Each Ct value was normalized to the Ct of the PolR2A gene and calculated as ΔCt [4], [25]. For data comparison between microarray and qRT-PCR, the Spearman's correlation coefficient (ρ) was used.

Comparing the qRT-PCR assays of HBRR versus UHRR published in MAQC [4], 996 genes out of 1,045 genes cited in the publication matched with gene probes carried on the 3D-Gene™ Human Oligo chip 25K. Following the MAQC protocol, genes detected in at least three of the five replicate samples from both assays were used for the analysis.

## Supporting Information

Table S1 ΔCt values of 42 genes detected from four human derived RNA samples analyzed by qRT-PCR.
(DOC)

Table S2 Accession numbers of all microarray data analyzed.
(DOC)
