# Supplementary material for: Use of Non-Amplified RNA Samples for Microarray Analysis of Gene Expression
Source: PLoS One. 2012 Feb 15;7(2):e31397. doi: 10.1371/journal.pone.0031397 (PMC3280296; doi:10.1371/journal.pone.0031397)
Supplement: Table S1 — ΔCt values of 42 genes detected from four human derived RNA samples analyzed by qRT-PCR. (DOC) [file pone.0031397.s001.doc]

|  | **Gene ID** | **3D-Gene^TM^Probe ID** | **ΔCt** | | | |
| --- | --- | --- | --- | --- | --- | --- |
|  |  |  | **UHRR** | **HBRR** | **Breast** | **Colon** |
| 1 | HYAL2 | H200006333 | -1.1 | -3.1 | -2.1 | -2.0 |
| 2 | FABP1 | H200021228 | 0.7 | -8.0 | -2.0 | 4.8 |
| 3 | ASNS | H200006172 | 0.1 | 0.1 | -3.2 | -1.8 |
| 4 | C20orf19 | H200020388 | -2.2 | -2.9 | -3.1 | -1.1 |
| 5 | CPS1 | H300003568 | 1.2 | -5.1 | -4.0 | -6.1 |
| 6 | P4HA2 | H200000807 | -5.2 | -7.7 | -6.4 | -5.4 |
| 7 | NEK6 | H300018986 | -0.9 | -2.0 | -2.2 | -2.0 |
| 8 | COL5A1 | H200013568 | -3.5 | -7.9 | -2.0 | -2.0 |
| 9 | GSTO2 | H300005421 | -5.7 | -5.5 | -3.3 | -5.0 |
| 10 | UGCGL2 | H300019551 | -0.7 | -2.9 | -2.2 | -2.1 |
| 11 | PRSS8 | H200006201 | -4.2 | -8.9 | -3.1 | -4.0 |
| 12 | ABCC5 | H300007492 | -2.7 | -1.8 | -3.3 | -3.0 |
| 13 | SYT3 | H200005590 | -7.2 | -3.0 | -4.0 | -5.0 |
| 14 | FBXO45 | H200001341 | 0.0 | -2.0 | -2.9 | -3.0 |
| 15 | GAPD | H200007830 | 6.6 | 5.1 | 2.8 | 3.0 |
| 16 | APOE | H200007822 | 1.2 | 2.1 | 1.7 | -0.1 |
| 17 | ENO1 | H200016354 | 5.7 | 3.0 | 2.7 | 2.4 |
| 18 | SOX9 | H200000590 | -2.0 | -2.1 | -1.0 | -1.8 |
| 19 | TTR | H200014619 | -0.3 | 2.0 | -9.0 | -4.2 |
| 20 | PGC | opHsV0400004806 | -6.7 | -1.9 | -1.5 | -0.6 |
| 21 | FN1 | H300022190 | 3.8 | -0.9 | 0.0 | 1.9 |
| 22 | MAGEA1 | H200005801 | -1.6 | -0.6 | -2.3 | -2.3 |
| 23 | RAB31 | H200015253 | 0.3 | -0.1 | 0.1 | 0.9 |
| 24 | TUBB6 | H200017078 | -0.7 | -3.8 | -1.1 | -2.0 |
| 25 | VCL | H200006081 | 0.8 | -1.1 | 1.0 | 1.7 |
| 26 | LRRC7 | H200007259 | -6.1 | -0.9 | -7.0 | -6.9 |
| 27 | NGB | H300009512 | -10.2 | -3.6 | -1.9 | -5.9 |
| 28 | PRKCG | H200000697 | -7.1 | -0.1 | -10.7 | -3.6 |
| 29 | PPFIA2 | H300009140 | -8.3 | -0.1 | -6.7 | -5.9 |
| 30 | SPOCK3 | H200007439 | -8.7 | 0.1 | -10.8 | -6.0 |
| 31 | ST18 | H200013768 | -8.8 | 0.1 | -11.3 | -8.0 |
| 32 | ELAVL | H200006039 | -9.4 | 0.0 | -9.8 | -4.0 |
| 33 | CA10 | opHsV0400003490 | -9.7 | 1.0 | -10.4 | -5.9 |
| 34 | ACRV1 | opHsV0400004233 | -11.1 | -1.1 | -7.0 | -5.4 |
| 35 | ANGPTL5 | opHsV0400002784 | -8.1 | -10.5 | -5.6 | -6.9 |
| 36 | HMCN1 | H300022168 | -2.3 | -7.8 | -2.1 | -3.1 |
| 37 | PLXNA2 | opHsV0400001198 | -3.7 | -1.0 | -4.0 | -2.8 |
| 38 | AKAP1 | H300019667 | -0.7 | -1.1 | -1.2 | 0.1 |
| 39 | STAU1 | H300019063 | 1.4 | 1.1 | 0.5 | 1.3 |
| 40 | ARS2 | opHsV0400004013 | 0.6 | -1.2 | -1.3 | -0.8 |
| 41 | CKAP4 | H300019430 | 0.8 | -1.4 | -1.0 | 0.3 |
| 42 | SLC2A3 | opHsV0400000011 | 1.3 | -0.1 | -2.5 | 0.2 |

**ΔCt was calculated from each Ct value normalized by the Ct of the PolR2A gene.*
