# Supplementary material for: Use of Non-Amplified RNA Samples for Microarray Analysis of Gene Expression
Source: PLoS One. 2012 Feb 15;7(2):e31397. doi: 10.1371/journal.pone.0031397 (PMC3280296; doi:10.1371/journal.pone.0031397)
Supplement: Table S2 — Accession numbers of all microarray data analyzed. (DOC) [file pone.0031397.s002.doc]

| GSE | GPL | Sample | Method | Accession |
| --- | --- | --- | --- | --- |
| GSE30945 | GPL5639 | UHRR | Non-Amp | GSM767246 |
|  |  | UHRR | Non-Amp | GSM767247 |
|  |  | UHRR | Non-Amp | GSM767248 |
|  |  | UHRR | Non-Amp | GSM767249 |
|  |  | UHRR | Non-Amp | GSM767250 |
|  |  | UHRR | 1xAmp | GSM767251 |
|  |  | UHRR | 1xAmp | GSM767252 |
|  |  | UHRR | 1xAmp | GSM767253 |
|  |  | UHRR | 1xAmp | GSM767254 |
|  |  | UHRR | 1xAmp | GSM767255 |
|  |  | UHRR | 2xAmp | GSM767256 |
|  |  | UHRR | 2xAmp | GSM767257 |
|  |  | UHRR | 2xAmp | GSM767258 |
|  |  | UHRR | Non-Amp | GSM767259 |
|  |  | UHRR | 1xAmp | GSM767260 |
|  |  | UHRR | 2xAmp | GSM767261 |
|  |  | HBRR | Non-Amp | GSM767262 |
|  |  | HBRR | Non-Amp | GSM767263 |
|  |  | HBRR | 1xAmp | GSM767264 |
|  |  | HBRR | 1xAmp | GSM767265 |
|  |  | HBRR | 2xAmp | GSM767266 |
|  |  | Breast | Non-Amp | GSM767267 |
|  |  | Breast | 1xAmp | GSM767268 |
|  |  | Breast | 2xAmp | GSM767269 |
|  |  | Colon | Non-Amp | GSM767270 |
|  |  | Colon | 1xAmp | GSM767271 |
|  |  | Colon | 2xAmp | GSM767272 |

*Abbreviation: UHRR: Universal Human Reference RNA, HBRR: Human Brain Reference RNA, Non-Amp: Non-amplification method, 1xAmp: 1-round amplification method, 2xAmp: 2-round amplification method.
